# Supplementary material for: Associative linking for collaborative thinking: Self-organization of content in online Q&A communities via user-generated links
Source: PLoS One. 2024 Mar 11;19(3):e0300179. doi: 10.1371/journal.pone.0300179 (PMC10927134; doi:10.1371/journal.pone.0300179)
Supplement: S1 Appendix — (DOCX) [file pone.0300179.s001.docx]

**S1 Appendix.** **References for Stack Exchange questions mentioned in Figures 1 and 2**

1. Why is rum naval? [Internet]. History Stack Exchange. [cited 2022 Sep 2]. Available from: https://history.stackexchange.com/questions/26952/why-is-rum-naval

2. When passports/border checks became widespread? [Internet]. History Stack Exchange. [cited 2022 Sep 2]. Available from:

https://history.stackexchange.com/questions/17190/when-passports-border-checks-became-widespread

3. How did modern border security and crossing bureaucracy develop? [Internet]. History Stack Exchange. [cited 2022 Sep 2].

Available from: https://history.stackexchange.com/questions/23053/how-did-modern-border-security-and-crossing-bureaucracy-develop?noredirect=1&lq=1

4. How can I obtain Leontief and Cobb-Douglas production function from CES function? [Internet]. Economics Stack Exchange. [cited 2022 Sep 2]. Available from: https://economics.stackexchange.com/questions/361/how-can-i-obtain-leontief-and-cobb-douglas-production-function-from-ces-function

5. Is zero inflation desirable? [Internet]. Economics Stack Exchange. [cited 2022 Feb 9]. Available from: https://economics.stackexchange.com/questions/5861/is-zero-inflation-desirable
