# Supplementary material for: Associative linking for collaborative thinking: Self-organization of content in online Q&A communities via user-generated links
Source: PLoS One. 2024 Mar 11;19(3):e0300179. doi: 10.1371/journal.pone.0300179 (PMC10927134; doi:10.1371/journal.pone.0300179)
Supplement: Attachment — Submitted filename: Response to Reviewers.docx [file pone.0300179.s002.docx]

Dear Editor and Reviewers,

We would like to thank you for your time and effort in reviewing our work. We highly apricate your feedback and have done our best to integrate the helpful suggestions and use them in improving the paper and further clarifying it. The following revisions were made in response to your feedback:

|  | Comment/suggestion | Response |
| --- | --- | --- |
| General |  |  |
|  | In your Methods section, please include additional information about how your Stack Exchange dataset was collected and ensure that you have included a statement specifying whether the collection and analysis method complied with the terms and conditions for the source of the data. | Additional information and statement were added (p. 11) |
| Reviewer 1 |  |  |
|  | The results that the authors find are not surprising and totally in line with previous works. It is indeed largely known in network science that random networks do not present clustering or other topological structures that are usually found in real networks [1,2,3]. For this reason I find a bit banal hypotheses H1 and H2, there was no reason to expect a different result.  I want to highlight that I found nothing wrong in the article, it is just that it should not be presented as a new discovery but just an analysis on a new dataset that confirms results that have been previously found on several types of real networks. I suggest to reorganize the text and reformulate the scientific question as looking for a confirmation of known results in the Q&A network too. | Thank you for this opportunity to clarify an important point regarding the goal of the research.  The goal of this work what was to use this known quality of real networks to make the point that the linked posts do form information networks, and that this is enabled by the users' individual linking activity.  The hypotheses addressed the user generated links' ability to introduce organization to the otherwise fragmented knowledge within the environment and to highlight the role of crosslinks within this process.  To further clarify this point and address the properties of the cross-link networks within the context of real-world networks, we've revised the phrasing of the hypotheses (See p. 9-10) |
|  | line 121: it seems that a sentence’s end is missing | Thank you, this was a leftover from a previous version and was removed. |
|  | Line 152: “organic bottom-up means” —> “organic bottom-up mean” | Means as in *means to an end*. |
|  | - Line 140: The sentence “the network consists of content units composed of question posts and their corresponding answers, connected by cross-links embedded by participants within their answers.” is not clear enough to explain how networks are built because the cross-links are explained only later, in the Methodology section | This is an excellent point, the sentence was rephrased to address and explain cross-links explicitly:  *In our case, the network consists of nodes comprising content units of question posts and edges based on cross-links, the hyperlinks embedded by participants within their answers that refer to other content units within the same discussion.* |
|  | Suggested citations:  1] Newman M. Networks. Oxford university press, 2018. [2] Barabasi A. Network Science. Cambridge University Press, 2016. [3] Latora V., Nicosia V., and Russo G. Complex networks: principles, methods and applications. Cambridge University Press, 2017. | Thank you for your highly relevant suggestions. We've integrated both Newman (2018) and Latora et al. (2016) into the manuscript. Barabasi (2016) was already cited within the paper. The additions are as follows:  Newman was cited in the introduction and in the conclusion by integrating the cross-linked discussions into Newman's classification of "networks of information" . An important insight into the role played by communities within networks from the work by Latora et al. (2017) was integrated into the manuscript |
| Reviewer 2 |  |  |
|  | The article's abstract must reflect the literature article's recommendation | The abstract was revised, hopefully the revisions reflect your input. |
|  | The background is too lengthy. Try to compress it. | The background has been shortened |
|  | Elaborate the concept with suitable diagrams as visual things elaborate the concept far better than the textual. | We appreciate this input. We've added another figure to illustrate the concept of cross-links |
|  | Expand the scalability of the article by including the research gap with prior work | See revised version of the paragraph beginning on line 119 |
|  | Read and cite the following articles 1.“Work coordination and collaborative knowledge construction in a small group collaborative virtual task” 2.“Faraway, Not So Close: The Conditions That Hindered Knowledge Sharing and Open Innovation in an Online Business Social Network” | Thank you for the references, the papers have been integrated into the manuscript (p. 3 and p.5) |
