# Supplementary material for: Associative linking for collaborative thinking: Self-organization of content in online Q&A communities via user-generated links
Source: PLoS One. 2024 Mar 11;19(3):e0300179. doi: 10.1371/journal.pone.0300179 (PMC10927134; doi:10.1371/journal.pone.0300179)
Supplement: Attachment — Submitted filename: Response to reviwers.docx [file pone.0300179.s003.docx]

# Response to reviewers

We would first like to thank you all for taking the time and making the effort to review this paper. Your feedback has been instrumental. We realize that some major points within the manuscript were not clear enough and needed further clarification. Mainly, it is important to emphasize that this research aimed to enrich our understanding of the collective creation of knowledge, by addressing the largely overlooked perspective of the shared product of virtual knowledge collaborations as a wholistic unit. While this was assisted by network science tools, these were not the main thrust of the paper in terms of novelty. We set out to point to the emergent networked nature of collectively created organizing structures, based on shared linking, and not to create a mathematical model for this phenomenon. While this could be a fascinating direction for future work, it is beyond the scope of the current one. We believe that the current literature has not given enough attention to the concept of shared linking as a means of creating a collective networked product, and its significance as a networked phenomenon has to first be explored, introduced, and acknowledged, before further investigation. Accordingly, the paper's aims and scope were further clarified. See the revised abstract, introduction, and discussion sections.

See more detailed responses to points raised within the reviews in the following table:

| Suggestion | Response |
| --- | --- |
| Reviewer 1: |  |
| In my previous review, I highlighted that the results reported in this article do not appear as new nor surprising, they are actually quite expected. I hence suggested to make this more evident from the paper. The authors agreed with that, but the only change that the authors made with respect to my concern was to add a couple of sentences to hypotheses H1 and H2, while I would have expected a more substantial change in how the story is told. I suggest to modify the abstract and the introduction too in this direction. Also, it would be useful if the changes in the text were highlighted so as to easily identify them. | As you have pointed out, the findings regarding the topology of the cross-linked networks are not surprising given the assumption that these qualify as real-world networks. However, the main idea of this research is not to establish these well-known phenomena in the study of real-world networks but rather to harness them to demonstrate that these unique kinds of networks, which have not received much attention in the existing literature of virtual knowledge collaborations, can be regarded as a collective product in the form of a network. This had to first be established, and then further explored by examining the emergence of modularity, the emergence of organizing structures over time, and the qualitative insights regarding the relations between the network properties and concurrent real-world events. As this has not been clear enough in the previous versions of the manuscript, several changes were made, including a re-phrasing of the abstract and the research hypotheses in this direction (p. 12).  See the version with highlighted tracked changes, especially the abstract and the introduction sections. |
| Reviewer 3 |  |
| 1) In the point of view of network theory it is a "meta-population" (in the network theory jargon), since inside each sub-network exists a number of nodes associated with questions, answers and comments, and links among them. It was not take into account. With effect, it was handled simply as nodes connected by chance with other nodes, meaning random links among different contents. I do not think it is surprisingly or highlighted findings. The mapping of a real-network into a random one, in this context, do not correspond to the usual modeling coming from network science. 2) Why do not consider a cross-link, in the sense connecting different sub-networks, in the context of a "Network of networks"? It will sounds to be a more natural mapping, since the connectivity distribution of each sub-network seems to have relevant/substantial role in the receiving attention by participants to "create" a cross-link with another relevant sub-network. Thus, each layer, corresponding to a network with specific content, whereas a cross-link connects different layers. | As you correctly point out, the full network of content within the Q&A discussion indeed includes questions, answers, and comments, and so the cross-linked network could be viewed as a "network of networks" comprising the connected content units as an overarching network with the question-answers-comments components as sub-networks. However, these subnetworks are by design hierarchical tree-shaped networks, and so emergent topological structures, which were the focus of this work, cannot appear within them. This phenomenon occurs within the question layer, as questions, and not answers or comments, become linked. For this reason, the more simplified network of content units was fit for purpose. Future work could further dive into other properties such as the number of answers and comments as a metric for a content-unit's significance. This explanation has been added to the paper (see p.6 and p.9 in the tracked changes version).  As for the mapping of the network, the randomized simulations were not an attempt to find a mathematical equation describing the network. Their role was in creating an estimated sampling distribution, using Bootstrap, to form statistical confidence intervals for the randomly linked networks' metrics, which served as a basis for comparison with the real metrics. Each of these simulations included 1000 iterations, which were used for extracting the metrics and creating the estimated sampling distributions. Importantly, we would like to emphasize here that the current work was not aimed at creating a model of such a network but rather to establish the "networkedness" of the collective artifact, by demonstrating that it harbors common real-world network properties that point to self-organization, as opposed to a random formation. This point has been added to the paper, with a more detailed explanation of the statistical process which we agree was not explained properly in the previous version (see abstract, introduction and methodological sections). |
| 3) I do not believe if the concept of a "hub" can be used on a connectivity distribution where the major number of nodes has a single connection, whereas the so-called "hubs" has four links... In Methodology section are described eight web sites corresponding to eight domain (network) of contents. Nodes are related to questions, besides answers and comments posted in response to questions. I believe that a more interesting question should be "what is the mathematical description for the connectivity distribution of cross-links of a Q&A networked system?", for example. For example, let take this sentence:  "In all the networks, the most connected content units in the real graphs had significantly more connections than their counterparts in the randomized graphs."  I really do not understand the novel information that a magazine's reader can take from this... "The random modeling do not extract the fundamental mechanism of a Q&A real-network. So, its not random, because participants do not choose by chance what they will response, question or comment." | We acknowledge your comment on the use of the concept of "hubs". Its use was an adaptation, as the cross-linked networks are very sparse, which is an artifact of the domain, and so what would commonly be referred to as "hubs" would be extremely rare, if possible at all. We added a paragraph that explains this and changed the wording accordingly (see p.9).  As for the mathematical equation, as mentioned, this was not the focus of the current work, and its significance is largely for literature on collective cognition and the collaborative creation of knowledge than for literature in network science. The main point was to demonstrate how individual activity that consists of sharing links results in changes to the overall structure of the network, which can be considered as organizing process. Future work could dive deeper to discover a mathematical description for this process, and perhaps use that to compare different discussions as there seems to be notable variance between communities which is worth exploring. |
| Another weak point: "These included the Economics network in which two large components of similar size were formed (one consisting of 46 nodes and the other of 43)"  I was expecting some statistical treatment to this lower number of elements. I my opinion, with no data treatment a comparison with an statistical ensemble, provided by 1000 elements (null model), is doubtful since no strong fluctuations can permeate the network realizations. Remember that for random networks the average degree is approximately k, but in the real-network counterpart not necessarely. | The description of the statistical procedure was revised and further clarified to address this point. The 1000 iterations of different randomly linked networks per each real network, which were similar in size and growth rate, network were used for extracting 1000 corresponding metrics for each of the networks, which act as a sample of randomly linked networks of similar size and growth rate. These 1000 "observations" for each of the metrics for each of the networks were used for creating an estimated sample distribution through bootstrap resampling which was used for creating a 99% confidence interval for each of the metrics. Falling beyond this interval demonstrates a high probability of not being the product of random linking, which strengthens the hypothesis that the cross-links introduce emergent order into the otherwise unorganized (and fragmented) discussions (see additions on pages 12 and 17). |
