# Supplementary material for: Associative linking for collaborative thinking: Self-organization of content in online Q&A communities via user-generated links
Source: PLoS One. 2024 Mar 11;19(3):e0300179. doi: 10.1371/journal.pone.0300179 (PMC10927134; doi:10.1371/journal.pone.0300179)
Supplement: Attachment — Submitted filename: Response to Reviewers.docx [file pone.0300179.s004.docx]

# Rebuttal letter

We would like to thank all of the reviewers who have taken their time and made the effort to read our work and make suggestions. Your contributions have been instrumental.

As reviewer 1 had no further comments in this round, reviewer 2 has not added any further comments after the first round, and reviewer 3 has indicated that they disagree with the concept of the paper, this response addresses the suggestions made by reviewer 4. Please see below:

Reviewer #4: The submission "Associative linking for collaborative thinking: self-organization of content in online Q&A communities via user-generated links" presents an analysis of the clustering, modularity, connectivity, and percolation of a suite of eight networks based on the StackExchange (SE) forums. These networks were defined on the set of threads--referred to here as "content units"--and edges were drawn between them based on whether or not a cross-link exists anywhere in the question, answer, or comments of the thread between thread x and thread y. The measurements on these networks are compared to what I would describe as a percolated hypercanonical Erdos-Renyi (ER) model in which the probability of an edge (or the number of links) is pulled from a distribution which matches the number of links created from each day in the data. This is not done in any sort of Monte-Carlo way, it is meant to match the exact percolation rate of each day seen in the data. The results show that the Stack Exchange networks exhibit clustering, max degree, nodes of degree >= 4, and largest component size to be outside of the 99% CI for 1000 runs of this percolated hypercanonical ER null model. They find also that the largest connected components exhibit high modularity and growth in the largest degree and number of triangles over time exceeds the rates seen in the null model.

It is my opinion that the strongest point of this article is that they have performed network analysis upon a knowledge-creating system which I have not seen an analysis of yet, and they do well to perform the relevant literature review.

However, there are a number of shortcomings in this paper which must be overcome for it to be ready to publish.

1a. The focus of the introduction and the motivation of this article seems to be to prove that these cross-link SE networks are "real networks." The authors state: "...this would enable us to reject the null hypothesis and to conclude that the cross-linked networks assume qualities that resemble real-world networks." [385-386] which is an odd hypothesis to have because there is no objective criterion for "real networks." A "network" is an ontological object and a "real network" would just be a real system which is examined through a graphical model. It seems that a lot of the conclusions about "emergent organization" rely on some proof that this cross-linked network is a "real network." It would be more salient to frame this as simply examining the features of this network (which is inherently a "real network" because it exists in the world and is being modeled graphically) and suggesting what that says about the system.

Authors' response:

Thank you for your input. Since as you mentioned, this type of information network has scarcely been addressed in the literature, we wanted to first establish that the collection of content-units (question and answer sets) connected by user-generated internal references constitutes as a network with a structure worth exploring. The wording was revised to better describe this.

2. This work has little to no basis in social-scientific theory about knowledge-contribution behavior, so I am uncertain as to what we have learned from this study besides that the cross-linked network likely has more internal structure than a random graph. The question remains: what are we to make of these result? In response to another reviewer who asked a similar question, I see that the response was that the goal is to bring attention “the concept of shared linking as a means of creating a collective networked product” but none of the papers cited (as far as I have seen) lend any meaning to this term “collective networked product” or its significance, let alone how these network properties map to this concept. The paper cites Barabasi’s 2003 paper to suggest that clustering implies organizing (also it may be worth considering Krioukov’s 2016 PRL paper) but this is very vague. Even a step forward to citing Newman’s definition in his 2005 textbook that ‘clustering implies transitivity’ would lend at least some theoretical advancement–-i.e. Cross-linking behaviors exhibit some transitivity.

Authors' response:

The theoretical concept behind the paper is highly connected to knowledge-contribution behavior: the internal complex structures present a form of self-organization (see for instance Heylighen (in Bates & Maack. eds), 2015). This structuring is suggested as form of knowledge integration that exceeds the integration created within the text or by other means of structuring virtual knowledge collaborative discussions such as tagging. This point has now been made much more salient within the text, especially in the introduction and theoretical concept sections.

3. There are a number of issues with the null model. It is a bit of an unusual null model to use because it seems to be a percolated G(n,p)/G(n,m) model with the link parameter matching the exact number of each day. The result of this would, ultimately however, still be a G(n,p) model with p=sum(edges)/sum(nodes) at the end. As this scales independently of the number of nodes, this is a dense model (see Def. 1.3 in Random Graphs and Complex Networks by Remco van der Hofstad) and thus I’m not sure this is the appropriate null model to measure the temporal dynamics of the network.

*We have given precise mathematical definitions for the notions of graph sequences being highly connected, small worlds, and scale free, extending earlier definitions in van der Hofstad (2010). Our definitions are based upon a summary of the relevant results proven for random graph models. We restrict ourselves to sparse random graphs, i.e., random graphs where the average degree remains bounded as the network size grows (recall Definition 1.3). In recent years, there has been an intensive and highly successful effort to describe asymptotic properties of graphs in the dense setting, where the average degree grows proportionally to the network size. This theory is described in terms of graph limits or graphons, which can be thought of as describing the limit of the rescaled adjacency matrix of the graph. The key ingredient in this theory is Szemer´edi’s regularity lemma (see Szemer´edi (1978)), which states roughly that one can partition a graph into several more or less homogeneous parts with homogeneous edge probabilities for the edges in between. See the book by Lov´asz (2012) for more details. We refrain from discussing the dense setting in more detail.*

Authors' response:

The number of both edges and nodes in the simulated graphs are identical to those of the original graphs, and were constructed as follows: the real graph's daily addition of nodes and edges was extracted, and the randomly-linked simulated graphs were constructed by gradually adding the daily number of nodes and edges while the structure of the graph remains. That is, nodes were added and then edges were randomly assigned on top of the existing structure from the previous simulated "day" (without repetition, only nodes that have not been linked could become linked). So, at the end of each simulated "day", there is a graph identical in a number of both nodes and links to the snapshot of the real graph on that day. On the "final day", the number of nodes and edges is identical to that of the real graph, and the average degree is of course identical. This was done to count for the greater chance of earlier nodes becoming connected, as the simulated graphs mimic the growth patterns of the real ones. This was iterated 1000 times so there eventually are 1000 simulated graphs for each community. So, the difference between the real graphs and their simulated counterparts is the assignments of edges, not their overall distribution which is the same. This creates ground for a comparison that singles out the contribution of the particular assignment of edges, to demonstrate that the way the edges are added has an organizing effect on the network as a whole, with more complex internal structures than would have been produced by assigning the edges randomly.

The main point is do demonstrate that the cross-links, originally designed for easing navigability between related content have effects in shaping the graph at the mezzo and macro levels.  *Other smaller things:*
a. Including triangles and clustering is redundant

Authors' response:

Global clustering is defined as the rate of closed triplets ("triangles") out of all possible triplets. i.e. two nodes connected to the same third node. Potentially, if these are very scarce, the rate of triangles might still be high. Forgoing the clustering coefficient, however, removes some of the context and the insight towards the concept of transitivity in the self-organization of the content, so we decided to keep both. This has now been added to be reflected in the text (table 3).

b. I'm uncertain as to why the terms "content units" and "focal points" are introduced and what purpose they serve. It seems that "content unit" refers to a thread in the forum, and “focal unit” is referring to a node of degree >= 4 but there is no justification as to why that was chosen, nor any theory behind it to suggest the significance of such in knowledge-sharing systems.

Authors' response:

The term "content unit" refers to a set of a question with its associated answers (if any) and comments (if any). Addressing the entire set of question + answers(+comments) stems from the perception of the entire set as a distinct unit of content within the network. These might be richer in case they contain more answers/ comments, but they stand alone as separate elements regardless. Their inner structure can be hierarchical, up to three layers (question-answers-comments). Amongst them, they can only be connected through a connective element, in this case, a cross-link (but studies have also looked at mutual tags as connectors, for instance (Dankulov et al., 2015)). Ye et al. (Ye et al., 2017) referred to these as knowledge-units. As part of the re-framing of the work, all references to these were altered to knowledge units – first in order to be consistent with existing work on similar environments, and second to reflect each question-answers-comments standalone value.

The term "focal point" of the network was used to describe a unit that has become relatively connected to other units. Within the entire dataset, hardly any nodes became connected enough to constitute as "hubs", but some nodes began to display potential of acting as local intersections that were at the crossroads of multiple knowledge units. Two indicators were used to identify the emergence of local focal points: 1. The degree of the most connected unit, and 2. The number of units with several connections (4 or above). While the number is somewhat arbitrary, it was designed to capture knowledge units that became connected to several other knowledge units, and so reflect an emergence of hierarchy, units that are more central than others within the collective knowledge product. This may be useful for navigation, or for extracting themes out of the graph by focusing on these nodes. This clarification was added to the manuscript.

Sometimes the word “sparse” gets used to seemingly describe how disconnected the network is. The ratio presented of links to nodes is actually dense enough to have one connected component and is dense in comparison to many other networks of real systems. In general, however, we measure sparsity and density within-component (so infinite distances get dropped).

Authors' response:

This has been corrected throughout the manuscript.

Broadly, my recommendation would be to zoom in and reframe the work so that the genuinely good results obtained can be understood through the lens of theory behind knowledge-sharing, instead of trying to develop novel theory about the relationships between network topology and epistemic knowledge-units alongside obtaining results.

Authors' response:

Thank you for this important suggestion. We have tried to incorporate your suggestions and shift the emphasis toward the role of link-sharing within the collaborative process.
